# Effect of active middle ear implant on auditory speech perception in individuals with ear malformation

**DOI:** 10.1590/2317-1782/e20240032en

**Published:** 2025-03-31

**Authors:** Eliane Aparecida Techi Catisquini, Mylena Malavazi Teixeira, Cláudia Daniele Pelanda Zampronio, Jerusa Roberta Massola Oliveira, Maria Fernanda Capoani Garcia Mondelli, Luiz Fernando Manzoni Lourençone, Rubens Vuono de Brito

**Affiliations:** 1 Divisão de Saúde Auditiva, Hospital de Reabilitação de Anomalias Craniofaciais, Universidade de São Paulo - Bauru (SP), Brasil.; 2 Programa de Residência Multiprofissional em Saúde Auditiva, Hospital de Reabilitação de Anomalias Craniofaciais, Universidade de São Paulo - Bauru (SP), Brasil.; 3 Departamento de Fonoaudiologia, Faculdade de Odontologia de Bauru, Universidade de São Paulo - Bauru (SP), Brasil.; 4 Curso de Medicina, Faculdade de Odontologia de Bauru, Universidade de São Paulo - Bauru (SP), Brasil.

**Keywords:** Hearing Loss, Hearing Aids, Ossicular Prosthesis, Speech Perception, Congenital Abnormalities

## Abstract

**Purpose:**

To verify the results of active middle ear implant on audibility and auditory speech perception in individuals with external and/or middle ear malformations.

**Methods:**

Primary, observational, retrospective study, through analysis of medical records of individuals with bilateral external and/or middle ear malformations, unilateral users of active middle ear implant. The data collected refer to auditory thresholds obtained through free-field audiometry and assessment of auditory speech perception - sentence recognition in silence and noise, in the following situations: without the implant, at the time of activation, in the first and in the third month of use.

**Results:**

Nine individuals were included in the study. The average age at the time of activation was 24.6 years (minimum 12 and maximum 40 years). Statistically significant improvement in auditory thresholds (p<0.05) and in the sentence recognition test in silence and noise (p<0.05) was observed at the time of activation. There was no significant difference between the evaluation situations after activation, indicating acclimatization by the user.

**Conclusion:**

The results of the active middle ear implant VSB (MED-EL) users on the audibility and auditory speech perception, in individual with external and/or middle ear malformation were better in the activation condition compared to the pre-surgical condition, maintaining stable over time; which reinforces its indication for this population.

## INTRODUCTION

Hearing losses differ in terms of type, degree and extent of impairment. When the use of drugs or surgical treatment is not amenable, the use of electronic devices is indicated, such as air conduction hearing aids, bone-anchored hearing aids, active middle ear prostheses and cochlear implants.

Active middle ear prostheses were designed to have the amplification mechanism surgically implanted in the middle ear as a common characteristic^(1)^. The prosthesis is made up of the following components: a microphone, processor, battery, receiver and transducer. They can be defined as fully implantable if all components are placed under the skin, or semi-implantable if only the receiver and transducer are implanted.

Several models of active middle ear prosthesis have become available over time, such as the fully implantable: Carina (Cochlear, Australia) and Esteen (Envoy Medical, USA); and semi-implantable: Vibrant Soundbridge (MED-EL, Austria), Codacs Direct Acoustic Cochlear Stimulation Implant (Cochlear, Australia) and Maxum (Ototronix Corporation, USA)^(2,3)^.

Specifically, the Vibrant Soundbridge (VSB) from the company MED-EL, Innsbruck, Austria, is considered a semi-implantable active middle ear prosthesis initially developed for individuals with mild to severe sensorineural hearing loss. With technological and surgical improvements, conductive and mixed losses could also be covered by this device.

**Figure 1:**
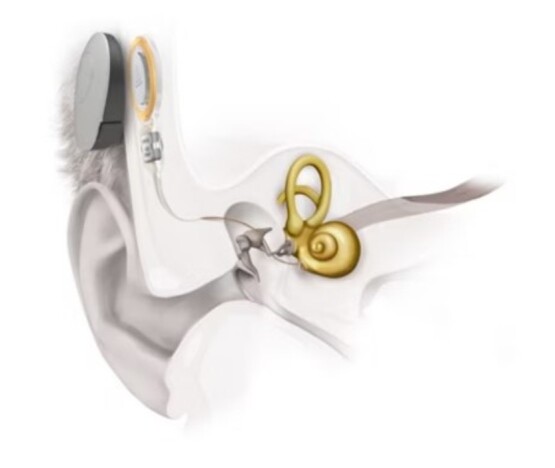
Illustration of the Vibrant Soundbridge active middle ear prosthesis (MED-EL)

**Figure 2:**
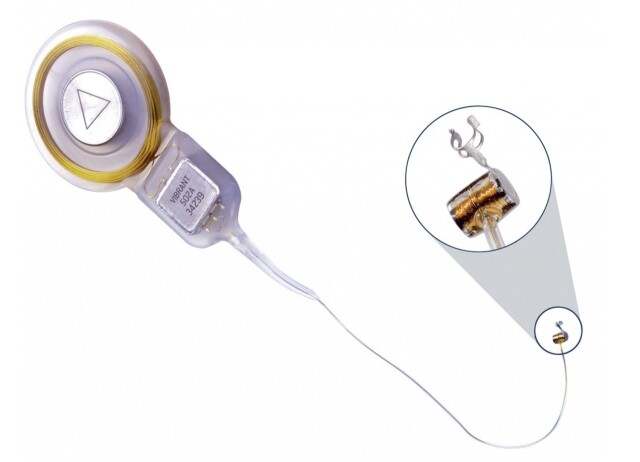
Illustration of the VORP internal component of the Vibrant Soundbridge active middle ear prosthesis (MED-EL)

The VSB (MED-EL) is made up of two parts: the external component called the sound processor which contains: a microphone, processor and battery; and the vibrating ossicular prosthesis (VORP) internal component, surgically implanted which contains: the receiver, the conductive wire and the electromagnetic transducer - floating mass transducer (FMT). The sound signals are captured by the microphone, converted into electrical energy, digitally processed and sent transcutaneously to the VORP (Figures 1 and 2)^(4)^.

The electrical energy is then converted into mechanical vibrations by the FMT, which can be coupled to the long or short process of the anvil, the base of the stirrup or directly to the round or oval window^(2,5)^. The FMT mechanically moves the structures of the middle ear and, thus, vibrations are transmitted to the cochlea. With the stimulation of hair cells, the electrical signal generated is sent to the cerebral cortex, favoring the understanding of speech sounds^(6,7)^.

The prosthesis is indicated for individuals who do not benefit from or are unable to use an air conduction hearing aid, such as those who have anatomical changes in the external and/or middle ear, infectious processes in the external auditory canal or limitation of the sound amplification provided by the hearing aid. The sound processor is magnetically maintained in the temporal region, and there is no occlusion of the external auditory canal. Since the internal component remains fixed and does not depend on the growth of the skull, the surgery can even be performed on children from the age of five^(8)^. When considering that mechanical stimulation occurs ipsilaterally, implantation can occur unilaterally or bilaterally without depending on the symmetry of bone conduction audiological thresholds as in bone-anchored hearing aids.

Researchers indicate the advantages of using this prosthesis when compared with sound amplification obtained through air conduction, providing the possibility of greater amplification without the occurrence of distortion, better sound quality, absence of the occlusion effect, lower risk of acoustic feedback and better results in speech recognition, even in situations of competitive noise^(7,9,10)^. In addition to the aforementioned advantages, since it is a transcutaneous prosthesis, fewer skin complications are observed compared to the percutaneous bone conduction system^(11)^.

In Europe and the United States, surgery to implant the VSB (MED-EL) occurred between 1998 and 2000^(5,11-13)^. In Brazil, it was only in 2010 that the surgery was regulated by the National Health Surveillance Agency; however, the prosthesis is not covered by the Unified Health System. Although the surgical procedure is safe, it requires qualified and experienced medical staff^(13,14)^. The prosthesis was initially only recommended for individuals over 18 years of age; however, with the improvement of surgical techniques, new transducer coupling options and satisfactory results observed, the indication was extended to children aged five years and over^(13,15,16)^.

The indication of an active middle ear prosthesis can be carried out when the individual does not benefit from or does not have favorable anatomical conditions for the adaptation of the air conduction hearing aid and depends on previously established medical and audiological criteria. The audiological criteria established by the manufacturer for the indication of the VSB (MED-EL) for sensorineural losses is determined by the air conduction threshold, which must be up to 65-85 dBHL; and through the bone conduction thresholds in cases of conductive and mixed losses whose thresholds must be up to 45-65 dBHL. To analyze the indication, frequencies from 500 to 4 kHz are considered^(6)^.

In individuals with external ear and/or middle ear malformations, such as microtia/atresia, hearing loss is generally observed and treatment options include air conduction hearing aid adaptation, bone conduction hearing aids and active middle ear prosthesis^(5)^. The definition of the treatment must be carried out by a team of professionals from related areas, such as the otorhinolaryngologist and the speech therapist with expertise in the area, and the decision must be shared with the individual in question^(13)^.

In 2018, a group of European researchers proposed some recommendations for developing a minimum protocol for working with an active middle ear prosthesis^(17)^; however, in Brazil, there are still no recommendations proposed by scientific entities.

There are several studies in the international literature^(5,14,16,18-22)^ that prove the effectiveness of active middle ear prostheses on audibility and, consequently, on the auditory perception of speech in individuals with external and/or middle ear malformations; however, there is a lack of national research that aims to discuss user performance and the results obtained with this device.

In view of the above, in order to contribute to the development of indication and monitoring protocols, the present study aimed to longitudinally verify the results of the active middle ear prosthesis on audibility and auditory speech perception in individuals with external and/or middle ear malformations.

## METHODS

The present is a study with a primary, observational and retrospective design, approved by the Research Ethics Committee under number 4,181.258. Data collection was carried out through documentary analysis of medical records of patients enrolled in the Hearing Health Division of the Hospital for Rehabilitation of Craniofacial Anomalies at the University of São Paulo, with the informed consent form (ICF) waived. Secondary data were collected using the Tasy hospital management software system from September to November 2020.

The established eligibility criteria inecluded: individuals with external and/or middle ear malformation, regardless of gender and age, who had bilateral conductive or mixed hearing loss and underwent surgery for the unilateral implantation of the Vibrant Soundbridge active middle ear prosthesis (MED-EL, Innsbruck, Austria), Amadé Hi model sound processor; effective users of the prosthesis for a period equal to or greater than 8 hours/day, self-reported and duly documented in the medical record.

The established exclusion criteria included: incomplete results regarding free-field audiometry and the assessment of auditory speech perception in some of the stages analyzed: pre- and post-surgery.

The service has a standardized clinical protocol for evaluating candidates and monitoring users of semi-implantable hearing aids. During the research period, the pre-surgical protocol for active middle ear prosthesis included evaluation without the hearing device, imaging tests and advice from the interdisciplinary team (ENT doctor, speech therapist, social worker and psychologist).

The VSB (MED-EL) was the prosthesis chosen among other possibilities, such as bone conduction by an interdisciplinary team considering the individual's otological needs and their consent. It is worth mentioning that the prosthesis was acquired through a research project, and the surgery was carried out by the hospital's own medical team with no financial support from the manufacturing company.

The information collected included: tonal thresholds obtained in a free field with the warble modulated tone at frequencies of 0.5, 1, 2, 3 and 4 kHz, according to the protocol established by the service; and the assessment of auditory speech perception - sentence recognition threshold in situations of silence and noise (signal/noise ratio), obtained through the use of lists of recorded sentences^(23)^. The procedures were carried out in an acoustic booth using a two-channel audiometer, the Madsen Astera 2 – Otometrics model calibrated in dBHL. The loudspeaker was positioned at 0° azimuth and one meter away from the individual.

To obtain the sentence recognition threshold in silence (SRTS), the ascending-descending technique^(24)^ was used, with the first sentence presented at an intensity of 65 dBHL without the processor and 40 dBHL with the processor turned on. For each correct sentence, the intensity was decreased in 4 dB steps until an error occurred. From this intensity, 2 dB increases were offered until a new correct sentence was observed, and so on, until the list of 10 sentences was completed. The presentation intensities of the sentences were noted during the test. The SRTS was calculated by averaging the intensity of sentence presentation starting from the first incorrect sentence.

To determine the sentence recognition threshold in noise (LRSR), the same technique was used with the presentation of the initial sentence at 65 dBHL and the competitive noise set at 60 dBHL (initial signal/noise ratio of +5 dB), with and without the sound processor. The final signal-to-noise ratio (S/N) was obtained by subtracting the LRSR from the fixed noise intensity used during the test (60 dBHL).

Data were collected in the pre-surgical stage without the use of hearing aids, considering the last available assessment and in the post-surgical stage at three moments: at activation, in the first month (post 1) and in the third month (post 2) of use of the active middle ear prosthesis.

In the descriptive analysis of the data, measures of central tendency (mean and median) and variability (standard deviation and 1st and 3rd quartile) were used. The Shapiro Wilk test was applied to verify the normality distribution of the data. For the inferential analysis when comparing the tonal thresholds in the conditions with and without the active middle ear prosthesis in the three evaluation moments, the non-parametric Friedman test was used, considering the first quartile (25%), the median (50%) and the third quartile (75%), since the majority of the sample did not present a normal distribution of data. Upon the presence of statistical significance, the Tukey´s multiple comparison test was used to indicate in which situation the significance occurred. For the inferential analysis of the SRTS and the S/N ratio, the analysis of the data distribution showed normality, and the ANOVA test was used with description of the mean and standard deviation and, subsequently, the Tukey´s test (SRTS) and the Brown Forsythe test (S/N) were applied to indicate situations of statistical significance. For all analyses, a significance level of 5% was considered.

## RESULTS

According to the eligibility criteria, 15 clinical records of individuals with bilateral external and/or middle ear malformations, users of the VSB active middle ear prosthesis (MED-EL), were selected. Of these, seven were excluded due to their failure to attend the institution to have the procedures reapplied three months after activating the external component.

Therefore, nine individuals were included in the study, unilateral users of the VSB prosthesis (MED-EL), and the Amadè Hi model sound processor whose demographic and audiological data are presented in Table 1.

**Table 1:** Demographic and audiological data of the participants

Variable		
Age	Mean ± SD	24.3 ± 10.5
	Extension	12 - 40
Gender	Feminine n (%)	6 (66.6)
	Masculine n (%)	3 (33.3)
Implanted side	Right n (%)	4 (44.4)
	Left n (%)	5 (55.5)
Degree of implanted ear hearing loss (dBHL)	Mild (26 – 40) n (%)	0
	Moderate (41 – 60) n (%)	6 (66.6)
	Severe (61 – 80) n (%)	3 (33.3)
	Profound (> 81) n (%)	0
Degree of contralateral ear hearing loss (dBHL)	Mild (26 – 40) n (%)	0
	Moderate (41 – 60) n (%)	7 (77.7)
	Severe (61 – 80) n (%)	2 (22.2)
	Profound (> 81) n (%)	0
Type of implanted ear hearing loss	Conductive n (%)	6 (66.6)
	Mixed n (%)	3 (33.3)
	Sensorineural n (%)	0
Type of contralateral ear hearing loss	Conductive n (%)	7 (77.7)
	Mixed n (%)	2 (22.2)
	Sensorineural n (%)	0
Average implanted ear BC hearing threshold* (dBHL)	Mean ± SD	9.2 ± 7.6
	Extension	3.8 - 25
Average contralateral ear BC hearing threshold^*^ (dBHL)	Mean ± SD	8.1 ± 5.5
	Extension	2.5 - 20
External and/or middle ear malformation	Non-syndromic etiology n (%)	9 (100)
	Syndromic etiology n (%)	0

* Average audiometric threshold at frequencies of 500, 1k, 2k and 4 kHz

Caption: BC = bone conduction; dBHL = decibel hearing level; SD = standard deviation

The average age at the time of the sound processor activation was 24.3 years (±10.5). Regarding the type, six individuals presented conductive hearing loss in the implanted ear and the average bone thresholds from 0.5 to 4 kHz ranged from 3.8 to 7.5 dBHL; the others had mixed hearing loss whose average ranged from 8.8 to 25 dBHL.

It is important to highlight that no individual in the present study presented pre or post-surgical complications after the implantation of the internal component, and the activation (programming) of the sound processor was carried out after evaluation and approval by the medical team. In our service, activation is carried out by the audiologist in the second month after the implantation of the internal component, following a standardized assessment protocol which allows auditory abilities, as well as individual needs regarding programming are checked longitudinally.

The sound processor programming settings were obtained using the Connexx software (Sivantos), inserted into the NOAH platform from the realization of the vibrogram – research of tonal thresholds at frequencies from 0.5 to 6 kHz through the sound processor which directly stimulates the FMT transducer. To adjust the processor, the values obtained in the vibrogram are used, as well as the individual's preference. The frequency range of the Amadè Hi model sound processor extends from 0.25 to 8 kHz, with a maximum gain of 54 dB according to the manufacturer's presentation.

**Figure 3:**
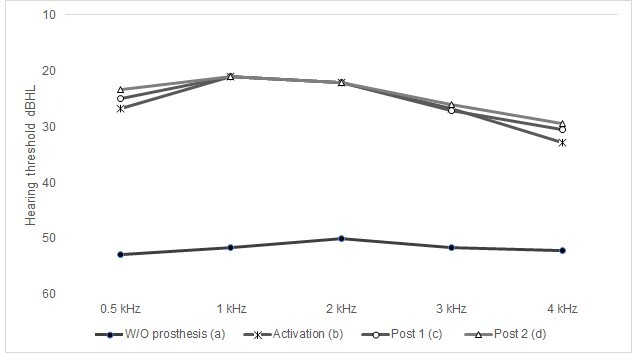
Average free-field tonal thresholds obtained at frequencies from 0.5 to 4 kHz without and with the active middle ear prosthesis in the three evaluation moments: activation, post 1 and post 2. Statistical significance (p<0.05): statistically significant for the condition without prosthesis (a) with the conditions activation (b), post 1 (c) and post 2 (d)

Figure 3 shows the average free-field tonal thresholds (dBHL) obtained at frequencies from 0.5 to 4 kHz with and without the active middle ear prosthesis in the three evaluation moments: activation, post 1 and post 2.

Caption: dBHL = decibel hearing level: W/O: without

The descriptive analysis of the results of free-field auditory thresholds revealed that, for a total of 9 individuals using the unilateral VSB prosthesis (MED-EL), the average frequency ranged from 0.5 to 4 kHz was 51.7 dBHL (± 9.9) in the pre-surgical condition. On the other hand, in the activation condition, it was observed that the average was 25.7 dBHL (±5.1), corresponding to an improvement of 26 dBHL. In post 1, the average was 24.9 dBHL (± 5.3) and, in post 2, the average was 23.8 (±5.0). In the analysis, stabilization of the results of the average auditory thresholds in a free field was noted between the activation, post 1 and post 2 conditions.

For the inferential analysis, the results showed a statistically significant improvement (p<0.05) in the activation of tonal thresholds obtained in a free field at most frequencies (0.5 to 3 kHz), in relation to the condition without the active prosthesis. middle ear VSB (MED-EL) (Figure 3 and Table 2)^(25)^.

**Table 2:** Statistical analysis when comparing tonal thresholds in free field and auditory speech perception without and with the active middle ear prosthesis in the three evaluation moments: activation, post 1 and post 2

	0,5k	1k	2k	3k	4k	SRTS	S/N
W/O VSB x Activation	0.031^*^ (a)	0.006*(a)	0.006*(a)	0.006*(a)	0.102	0.001*(a)	0.002**(a)
W/O VSB x Post 1	0.002*(b)	0.006*(b)	0.006*(b)	0.010*(b)	0.008*(b)	0.001*(b)	0.001**(b)
W/O VSB x Post 2	0.002*(c)	0.006*(c)	0.006*(c)	0.010*(c)	0.001*(c)	0.001*(c)	0.005**(c)
Activation x Post 1	0.844	1.000	1.000	0.998	0.798	0.832	0.999
Activation x Post 2	0.844	1.000	1.000	0.998	0.519	0.930	0.967
Post 1 x Post 2	1.000	1.000	1.000	1.000	0.968	0.995	0.932

* Tukey test (p<0.05) and Brown-Forsythe test (p<0.05)

Statistical significance (p<0.05): (a) statistically significant - condition without Vibrant Soundbridge x activation for 0.5, 1, 2 and 3 kHz, SRTS and S/N: (b) statistically significant - condition without Vibrant Soundbridge x post 1 for 0.5, 1, 2, 3 and 4 kHz, SRTS and S/N: (c) statistically significant - condition without Vibrant Soundbridge x post 2 for 0.5, 1, 2, 3 and 4 kHz, SRTS and S/N

Caption: SRTS = sentence recognition threshold in silence; S/N = signal-to-noise ratio: W/O VSB = without Vibrant Soundbridge

Table 2 shows the statistical analysis when comparing the tonal thresholds in a free field (dBHL) and auditory speech perception with and without the active middle ear prosthesis in the three evaluation moments: activation, post 1 and post 2.

When analyzing the 4 kHz frequency, an improvement was observed between the hearing threshold observed in the situation without the prosthesis (55 dBHL ±9.7) and the moment of activation (30 dBHL ±15.4); however, a significant difference was observed between the situations without the prosthesis and post 1, which remained stable in post 2 (Figure 3 and Table 2). This finding suggests better audibility after the period of auditory stimulation.

Furthermore, in Figure 3, it was possible to observe that the tonal thresholds obtained in a free field at the time of activation of the prosthesis remained stable in post 1 and post 2 for frequencies from 0.5 to 3 kHz.

**Figure 4:**
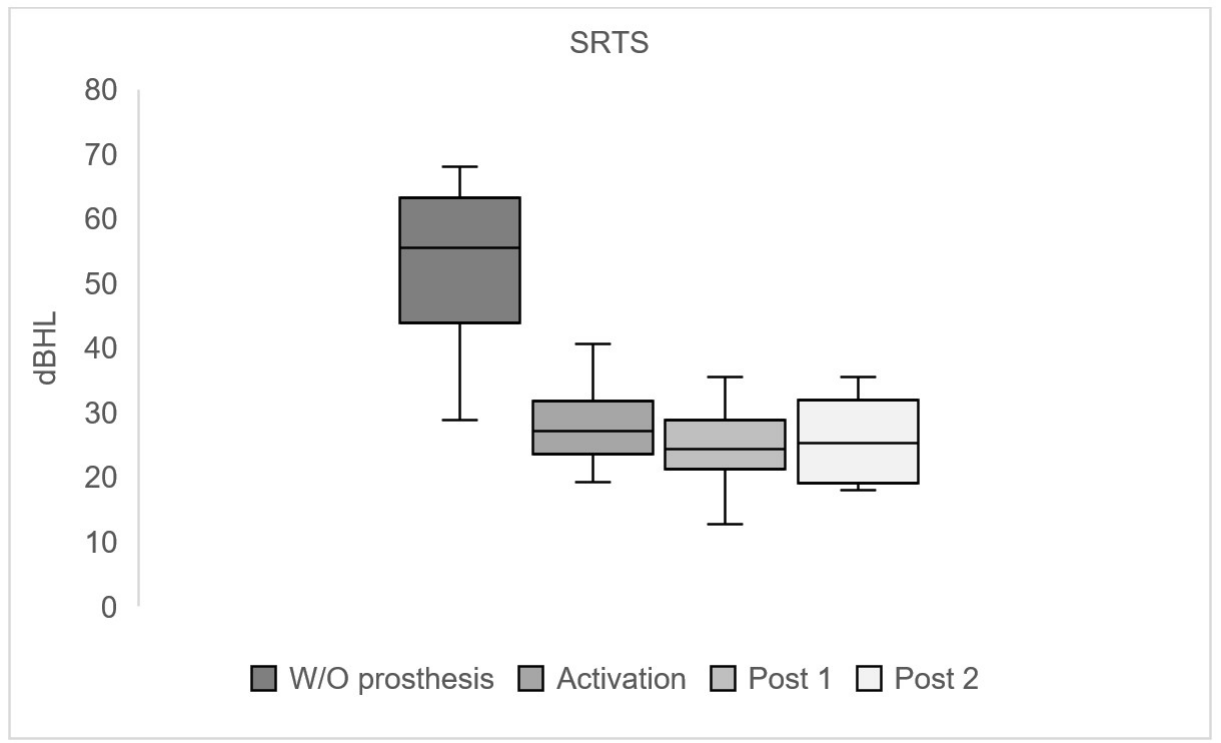
Median sentence recognition thresholds in silence (dBHL) without and with the active middle ear prosthesis in the three evaluation moments: during activation, post 1 and post 2

The analysis of the results with the hypothesis tests applied in the evaluation of auditory perception of speech in silence demonstrated a significant improvement when compared to situations without the prosthesis and at the time of its activation (p<0.05). Similar to what was observed with tonal thresholds in a free field, after activation, the results became similar and did not show a significant difference (Figure 4 and Table 2).

Caption: dBHL = decibel hearing level; SRTS = sentence recognition threshold in silence; W/O = without

**Figure 5:**
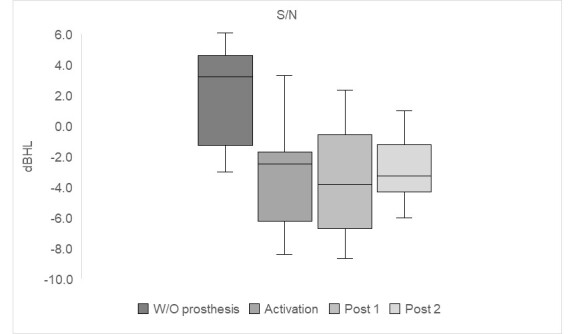
Median S/N ratio without and with the active middle ear prosthesis at the three assessment moments: at activation, post 1 and post 2

Figures 4 and 5 present, respectively, the median of sentence recognition thresholds in silence (dBHL) and the S/N ratio. The SRTS analysis revealed an improvement of 28.5 dBHL in activation and the S/N ratio, observed at 3.2 dB pre-surgery, reached a median of -2.5 dB in activation. It is important to highlight that when evaluating speech in noise, the lower the S/N value, the better the result obtained with the speech material. Over time, the results obtained in the speech tests remained stable at an equally significant level compared to the pre-surgical condition.

Caption: dBHL = decibel hearing level; S/N = signal-to-noise ratio; W/O = without

## DISCUSSION

With technological advances in recent years, individuals with external and/or middle ear malformations who are unable or do not benefit from air conduction amplification now have greater options for auditory rehabilitation with regard to semi-implantable hearing aids. This category includes bone conduction hearing aids and active middle ear hearing aids.

The Vibrant Soundbridge (MED-EL) active middle ear prosthesis, the object of the present study, was introduced on the market in 1996 and has stood out since its indication now covers, in addition to sensorineural hearing losses, conductive and mixed hearing losses^(26)^. With the internal transducer (FMT) coupling options, initially restricted to the ossicular chain, extending to the oval and round window, the prosthesis became a possibility for individuals with external and/or middle ear malformations^(22,27)^; however, the possible coupling site must be carefully evaluated through a previously performed imaging examination so that the surgeon can estimate the degree of the malformation and determine the possibilities of FMT coupling^(5)^.

The success of the surgery will depend on a stable connection between the transducer and the anatomical structure that allows mechanical vibration and favorable signal transmission at all frequencies throughout the cochlea^(5)^. Given the safety, effectiveness and stability of the device observed in longitudinal studies^(9,22,28,29)^, the VSB prosthesis (MED-EL) was approved for children aged five years and over^(13,15,16)^, which enabled the inclusion of four participants between 12 and 17 years old in the present study, in whom no pre or post-surgical complications were observed.

For the audiological evaluation of the result of auditory rehabilitation provided by the VSB (MED-EL), parameters related to audibility and auditory speech perception need to be considered and established through a clinical protocol that favors the monitoring of individuals regarding the use, benefit of technology and programming adjustment needs.

The tonal thresholds observed in a free field with the prosthesis showed excellent audibility for the main frequencies evaluated right at the moment of activation, which continued throughout the post-surgical moments (Figure 1). These findings show that access to sounds is possible through the use of the prosthesis and favors the recovery of speech detection skills, essential for performance in speech recognition and understanding tasks and corroborates previous studies^(14,19,21)^.

Previous studies^(14,21,27)^ corroborate the improvement in tonal thresholds with the use of an active middle ear prosthesis right at the moment of processor activation and emphasize that such results are expected due to the vibration of the middle ear structures provided by surgical positioning, adequate FMT and directing the sound stimulus to the inner ear^(11,20,21,28)^, a factor that determines the importance of a well-trained and experienced medical team in otological surgeries^(14)^.

The improvement observed in the assessment of auditory speech perception through the sentence recognition threshold in silence upon activation and the stability of responses in post-surgical assessments also pointed to the effectiveness of the prosthesis (Figure 4 and Table 2). Studies that evaluated user performance with the active middle ear prosthesis over time revealed that the first substantial change in the results observed in tonal thresholds and sentence recognition thresholds occurred until the third month^(9,18)^. After this period, no significant changes were observed^(14,16,28-30)^, thus indicating the stability of the results.

Auditory speech perception was also assessed in competitive noise. The results pointed to the same pattern observed in the silent situation; that is, the individual's performance was better immediately after activating the sound processor (Figure 5), with a significant difference (p<0.05), which remained stable in the other evaluation moments, that is, in post 1 and post 2 (Table 2).

It is known that satisfactory results in auditory speech perception are related to better performance in communication situations, especially in noisy situations; a condition inherent to daily life situations which tends to favor the effective use of the hearing device and thus provide a better quality of life.

The stability of tonal thresholds obtained in a free field and in the evaluation of auditory speech perception carried out in the short and long term was verified in previous studies with the same prosthesis^(14,16,28-30)^. This stability can be justified by acclimatization since the connections reach the peak of maximum activity after stimulation in a short time with the optimization of the prosthesis, thus obtaining the desired benefit. Mechanical stimulation of the middle ear tends to favor neural plasticity, allowing the central auditory pathways to reorganize and provide positive effects on auditory abilities^(11,20,21)^.

The evaluation of the individual in the pre-surgical stage, at the activation of the prosthesis and longitudinally during the follow-up, requires a protocol previously established by the service in order to evaluate the audibility and auditory perception of speech in situations of silence and noise. The protocol established in our service aims to establish parameters for comparing results over time, monitoring the individual's evolution and verifying the need for processor adjustments in order to guarantee effective use and, thus, the achievement of the desired benefits.

The results presented here are in accordance with the literature^(5,16,22,27-30)^ since they reveal an improvement in audibility and auditory perception of speech with the prosthesis, even without auditory stimulation in the contralateral ear. The achievement of satisfactory results, observed longitudinally through a standardized clinical protocol, suggests that the VSB (MED-EL) active middle ear prosthesis is an effective option for individuals with external and/or middle ear malformations who cannot benefit from rehabilitation using air conduction hearing aids. The effective use of the prosthesis tends to favor the process of communication, socialization, academic performance, insertion and maintenance in the job market and, consequently, an improvement in quality of life.

## CONCLUSION

The results of the VSB (MED-EL) active middle ear prosthesis on audibility and auditory speech perception in individuals with external and/or middle ear malformations were better in the activation condition compared to the pre-surgical condition, maintaining stability over time, which reinforces its indication for this population.
