# Supplementary material for: Effect of active middle ear implant on auditory speech perception in individuals with ear malformation
Source: Codas. 2025 Mar 31;37(3):e20240032. doi: 10.1590/2317-1782/e20240032en (PMC11963885; doi:10.1590/2317-1782/e20240032en)
Supplement: Translation

# Translation: Efeito da prótese ativa de orelha média na percepção auditiva da fala em indivíduos com malformação de orelha

**DOI:** 10.1590/2317-1782/e20240032pt

**Authors:** Eliane Aparecida Techi Catisquini, Mylena Malavazi Teixeira, Cláudia Daniele Pelanda Zampronio, Jerusa Roberta Massola Oliveira, Maria Fernanda Capoani Garcia Mondelli, Luiz Fernando Manzoni Lourençone, Rubens Vuono de Brito

**Affiliations:** 1 Divisão de Saúde Auditiva, Hospital de Reabilitação de Anomalias Craniofaciais, Universidade de São Paulo - Bauru (SP), Brasil.; 2 Programa de Residência Multiprofissional em Saúde Auditiva, Hospital de Reabilitação de Anomalias Craniofaciais, Universidade de São Paulo - Bauru (SP), Brasil.; 3 Departamento de Fonoaudiologia, Faculdade de Odontologia de Bauru, Universidade de São Paulo - Bauru (SP), Brasil.; 4 Curso de Medicina, Faculdade de Odontologia de Bauru, Universidade de São Paulo - Bauru (SP), Brasil.

**Keywords:** Perda Auditiva, Auxiliares de Audição, Prótese Ossicular, Percepção da Fala, Anormalidades Congênitas

## Abstract

**Objetivo:**

Verificar os resultados da prótese ativa de orelha média na audibilidade e na percepção auditiva da fala em indivíduos com malformação de orelha externa e/ou média.

**Método:**

Estudo primário, observacional, retrospectivo, por meio da análise de prontuários de indivíduos com malformação de orelha externa e/ou média bilateral, usuários unilaterais de prótese ativa de orelha média. Os dados coletados referem-se aos limiares auditivos obtidos por meio da audiometria em campo livre e avaliação da percepção auditiva da fala - reconhecimento de sentenças no silêncio e no ruído, nas seguintes situações: sem a prótese, no momento da ativação, no primeiro e no terceiro mês de uso.

**Resultados:**

Nove indivíduos foram incluídos no estudo. A idade média no momento da ativação foi de 24,6 anos (mínimo 12 e máximo 40 anos). Melhora estatisticamente significante dos limiares auditivos (p<0,05) e no teste de reconhecimento de sentenças no silêncio e no ruído (p<0,05) foi observada no momento da ativação da prótese. Não houve diferença significante entre as situações de avaliação após a ativação, indicando aclimatização pelo usuário.

**Conclusão:**

Os resultados da prótese ativa de orelha média VSB (MED-EL) na audibilidade e na percepção auditiva da fala em indivíduos com malformação de orelha externa e/ou média foram melhores na condição da ativação, em comparação à condição pré-cirúrgica, mantendo-se estável ao longo do tempo; o que reforça sua indicação para essa população.

## INTRODUÇÃO

As perdas auditivas diferenciam-se quanto ao tipo, grau e extensão do seu comprometimento. Quando não passíveis de tratamento medicamentoso ou cirúrgico, o uso de dispositivos eletrônicos é indicado, como o aparelho de amplificação sonora individual (AASI), as próteses auditivas ancoradas no osso (PAAO), as próteses ativas de orelha média e o implante coclear.

As próteses ativas de orelha média foram projetadas e desenvolvidas para terem como característica comum o mecanismo de amplificação implantado cirurgicamente na orelha média^(1)^. A prótese é composta pelos seguintes componentes: microfone, processador, bateria, receptor e transdutor. Elas podem ser classificadas em totalmente implantáveis, se todos os componentes estiverem sob a pele, ou semi-implantáveis, se apenas o receptor e o transdutor forem estiverem implantados.

Vários modelos de prótese ativa de orelha média foram disponibilizados ao longo do tempo, como as totalmente implantáveis: Carina (Cochlear, Austrália) e Esteen (Envoy Medical, USA); e as semi-implantáveis: Vibrant Soundbridge (MED-EL, Áustria), Codacs Direct Acoustic Cochlear Stimulation Implant (Cochlear, Austrália) e Maxum (Ototronix Corporation, USA)^(2,3)^.

Especificamente, a Vibrant Soundbridge (VSB), da empresa MED-EL, Innsbruck, Áustria, é considerada uma prótese ativa de orelha média semi-implantável desenvolvida, inicialmente, para indivíduos com perda auditiva sensorioneural de grau leve a severo. Com o aprimoramento tecnológico e cirúrgico, as perdas condutivas e mistas também puderam ser contempladas por este dispositivo.

A VSB (MED-EL) é composta por duas partes: o componente externo, denominado processador de som, que contém: microfone, processador e bateria; e o componente interno, Vibration Ossicular Prosthesis (VORP), cirurgicamente implantado, que contém: o receptor, o fio condutor e o transdutor eletromagnético - Floating Mass Transducer (FMT). Os sinais sonoros são captados pelo microfone, convertidos em energia elétrica, processados digitalmente e enviados, transcutâneamente, para o VORP (Figuras 1 e 2)^(4)^.

**Figura 1:** Ilustração da prótese ativa de orelha média Vibrant Soundbridge (MED-EL)

**Figura 2:** Ilustração do componente interno VORP da prótese ativa de orelha média Vibrant Soundbridge (MED-EL)

A energia elétrica é então convertida em vibrações mecânicas pelo FMT, que pode ser acoplado ao processo longo ou curto da bigorna, à base do estribo ou diretamente à janela redonda, ou oval^(2,5)^. O FMT movimenta mecanicamente as estruturas da orelha média e, assim, as vibrações são transmitidas à cóclea. Com a estimulação das células ciliadas, o sinal elétrico gerado é enviado ao córtex cerebral e, então, a compreensão dos sons da fala é favorecida^(6,7)^.

A prótese é indicada para indivíduos que não se beneficiam ou estão impossibilitados de utilizar o AASI por condução aérea, como aqueles que apresentam alterações anatômicas de orelha externa e/ou média, processos infecciosos do CAE ou limitação da amplificação sonora fornecida pelo AASI. O processador de som é mantido, magneticamente, na região temporal e não ocorre a oclusão do conduto auditivo externo (CAE). Como o componente interno permanece fixo e não depende do crescimento do crânio, a cirurgia pode ser realizada, inclusive, em crianças, a partir dos cinco anos^(8)^. Ao considerar que a estimulação mecânica ocorre ipsilateralmente, a implantação pode ocorrer uni ou bilateralmente, não dependendo da simetria dos limiares audiológicos de via óssea, como nas PAAO.

Os pesquisadores indicam as vantagens da utilização dessa prótese ao ser comparada com a amplificação sonora obtida por meio da condução aérea sendo a possibilidade de maior amplificação sem a ocorrência de distorção, melhor qualidade sonora, ausência do efeito de oclusão, menor risco de feedback acústico e melhores resultados no reconhecimento de fala, mesmo em situações de ruído competitivo^(7,9,10)^. Além das vantagens citadas, por se tratar de prótese transcutânea, menos complicações de pele são observadas em comparação ao sistema de condução óssea percutâneo^(11)^.

Na Europa e nos Estados Unidos, a cirurgia para a implantação do VSB (MED-EL) ocorre desde 1998 e 2000, respectivamente^(5,11-13)^. No Brasil, somente em 2010 a cirurgia foi regulamentada pela Agência Nacional de Vigilância Sanitária; todavia, a prótese não é contemplada pelo Sistema Único de Saúde. Apesar do procedimento cirúrgico ser seguro, requer equipe médica qualificada e experiente^(13,14)^. A prótese, inicialmente, era indicada apenas para indivíduos maiores de 18 anos; porém, com o aprimoramento das técnicas cirúrgicas, novas opções de acoplamento do transdutor e resultados satisfatórios observados, a indicação foi estendida para crianças a partir dos cinco anos^(13,15,16)^.

A indicação da prótese ativa de orelha média pode ser realizada quando o indivíduo não se beneficia ou não possui condições anatômicas favoráveis para a adaptação do AASI por condução aérea e depende de critérios médicos e audiológicos previamente estabelecidos. O critério audiológico estabelecido pela fabricante para a indicação da VSB (MED-EL) para perdas sensorioneurais é determinado por: via aérea, cujos limiares devem estar até 65-85 dBNA; e pela via óssea, nos casos de perdas condutivas e mistas, cujos limiares devem estar até 45-65 dBNA. Para a análise da indicação considera-se as frequências de 500 a 4 kHz.

Em indivíduos com malformação de orelha externa e/ou orelha média, como na microtia/atresia, a perda auditiva é geralmente observada e dentre as opções de tratamento estão a adaptação do AASI por condução aérea, a PAAO e a prótese ativa de orelha média^(5)^. A definição do tratamento deve ser realizada por uma equipe de profissionais de áreas afins, como o médico otorrinolaringologista e o fonoaudiólogo com domínio na área, e a decisão deve ser compartilhada com o indivíduo em questão^(13)^.

Em 2018, um grupo de pesquisadores europeus propôs algumas recomendações para a elaboração de protocolo mínimo para a atuação com prótese ativa de orelha média^(17)^; porém, no Brasil, ainda não há recomendações propostas por entidades científicas.

Existem vários estudos na literatura internacional^(5,14,16,18-22)^ que comprovam a eficácia da prótese ativa de orelha média na audibilidade e, consequentemente, na percepção auditiva da fala de indivíduos com malformação de orelha externa e/ou média; porém, há escassez de pesquisas nacionais que visem discutir o desempenho do usuário e os resultados obtidos com esse dispositivo.

Diante do exposto, a fim de contribuir na elaboração de protocolos de indicação e de acompanhamento, esse estudo teve por objetivo verificar, longitudinalmente, os resultados da prótese ativa de orelha média na audibilidade e na percepção auditiva da fala em indivíduos com malformação de orelha externa e/ou média.

## MÉTODO

Trata-se de um estudo com desenho primário, observacional e retrospectivo, aprovado pelo Comitê de Ética em Pesquisa sob o número 4.181.258. A coleta dos dados foi realizada por meio da análise documental de prontuários de pacientes matriculados na Divisão de Saúde Auditiva do Hospital de Reabilitação de Anomalias Craniofaciais da Universidade de São Paulo, sendo dispensado o Termo de Consentimento Livre e Esclarecido. Os dados secundários foram coletados por meio do sistema de *software* de gestão hospitalar Tasy, no período de setembro a novembro de 2020.

Os critérios de elegibilidade estabelecidos foram: indivíduos com malformação de orelha externa e/ou média, independentemente do gênero e idade, que apresentavam perda auditiva condutiva ou mista bilateral e realizaram a cirurgia para a implantação unilateral da prótese ativa de orelha média Vibrant Soundbridge (MED-EL, Innsbruck, Áustria), processador de som modelo Amadé Hi; usuários efetivos da prótese por período igual ou superior a 8h/dia, auto referido e devidamente documentado no prontuário.

Os critérios de exclusão estabelecidos foram: resultados incompletos referentes à audiometria em campo livre e a avaliação da percepção auditiva da fala em algumas das etapas analisadas: pré e pós-cirúrgica.

O serviço possui protocolo clínico padronizado para avaliação de candidatos e acompanhamento de usuários de próteses auditivas semi-implantáveis. No período de realização da pesquisa, o protocolo pré-cirúrgico para a prótese ativa de orelha média compreendia a avaliação sem o dispositivo auditivo, exames de imagem e aconselhamento da equipe interdisciplinar (médico otorrinolaringologista, fonoaudiólogo, assistente social e psicólogo).

A VSB (MED-EL) foi a prótese eleita, dentre outras possibilidades, como as de condução óssea, por equipe interdisciplinar, se considerando as necessidades otológicas do indivíduo e sua anuência. Vale ressaltar que a prótese foi adquirida por meio de projeto de pesquisa e a cirurgia realizada por equipe médica do próprio hospital, não havendo apoio financeiro da empresa fabricante.

As informações coletadas foram: os limiares tonais obtidos em campo livre, com o tom modulado *warble*, nas frequências de 0,5, 1, 2, 3 e 4 kHz, conforme protocolo estabelecido pelo serviço; e a avaliação da percepção auditiva da fala - limiar de reconhecimento de sentenças nas situações de silêncio e ruído (relação sinal/ruído), obtida por meio da utilização das listas de sentenças gravadas^(23)^. Os procedimentos foram realizados em cabine acústica, utilizando-se um audiômetro de dois canais, modelo Astera 2 Madsen - Otometrics, calibrado em dBNA. A caixa acústica foi posicionada a 0° azimute e a um metro de distância do indivíduo.

Para a obtenção do limiar de reconhecimento de sentenças no silêncio (LRSS), foi utilizada a técnica ascendente-descendente^(24)^, com a primeira sentença apresentada na intensidade de 65 dBNA, sem o processador, e 40 dBNA com o processador ligado. A cada sentença correta, a intensidade foi diminuída em passos de 4 dB até que ocorresse um erro. A partir dessa intensidade foram oferecidos acréscimos de 2 dB, até que uma nova sentença correta fosse observada, e assim sucessivamente, até completar a lista de 10 sentenças. As intensidades de apresentação das sentenças foram anotadas no decorrer do teste. O LRSS foi calculado pela média das intensidades de apresentação das sentenças a partir da primeira incorreta.

Para a determinação do limiar de reconhecimento de sentenças no ruído (LRSR) foi utilizada a mesma técnica, com a apresentação da sentença inicial em 65 dBNA e o ruído competitivo fixado em 60 dBNA (relação sinal/ruído inicial de +5 dB), sem e com o processador de som. A relação sinal/ruído (S/R) final foi obtida subtraindo-se o LRSR da intensidade de ruído fixo utilizada durante o teste (60 dBNA).

Os dados foram coletados na etapa pré-cirúrgica: sem o uso da prótese auditiva, sendo considerada a última avaliação disponível; e na etapa pós-cirúrgica em três momentos: na ativação, no primeiro mês (pós 1) e no terceiro mês (pós 2) de uso da prótese ativa de orelha média.

Na análise descritiva dos dados foram utilizadas a medida de tendência central (média e mediana) e de variabilidade (desvio padrão e 1º e 3º quartil). O teste de Shapiro Wilk foi aplicado para verificação da distribuição de normalidade dos dados. Para a análise inferencial, na comparação dos limiares tonais nas condições sem e com a prótese ativa de orelha média nos três momentos de avaliação, foi utilizado o teste não paramétrico de Friedman, considerando o primeiro quartil (25%), a mediana (50%) e o terceiro quartil (75%), visto que a maioria da amostra não apresentou distribuição normal de dados. Mediante a presença de significância estatística utilizou-se o teste de comparação múltipla Tukey para indicar em qual situação deu-se a significância. Para a análise inferencial do LRSS e da relação S/R a análise da distribuição dos dados acusou normalidade, assim foi empregado o teste ANOVA com descrição da média e desvio padrão e, subsequente, foi aplicado o teste Tukey (LRSS) e teste Brown-Forsythe (S/R) para indicar as situações de significância estatística. Para todas as análises foi considerado nível de significância de 5%.

## RESULTADOS

Conforme os critérios de elegibilidade, 15 prontuários de indivíduos com malformação de orelha externa e/ou média bilateral, usuários da prótese ativa de orelha média VSB (MED-EL), foram selecionados. Destes, sete foram excluídos em razão do não comparecimento à instituição para a reaplicação dos procedimentos após três meses da ativação do componente externo.

Sendo assim, foram incluídos no estudo nove indivíduos, usuários unilaterais da prótese VSB (MED-EL), processador de som modelo Amadè Hi, cujos dados demográficos e audiológicos estão apresentados na Tabela 1.

**Tabela 1:** Dados demográficos e audiológicos dos participantes

Variável		
Idade	Média ± DP	24,3 ± 10,5
	Extensão	12 - 40
Gênero	Feminino n (%)	6 (66,6)
	Masculino n (%)	3 (33,3)
Lado implantado	Direito n (%)	4 (44,4)
	Esquerdo n (%)	5 (55,5)
Grau da perda auditiva orelha implantada (dBNA)	Leve (26 – 40) n (%)	0
	Moderado (41 – 60) n (%)	6 (66,6)
	Severo (61 – 80) n (%)	3 (33,3)
	Profundo (> 81) n (%)	0
Grau da perda auditiva orelha contralateral (dBNA)	Leve (26 – 40) n (%)	0
	Moderado (41 – 60) n (%)	7 (77,7)
	Severo (61 – 80) n (%)	2 (22,2)
	Profundo (> 81) n (%)	0
Tipo de perda auditiva orelha implantada	Condutiva n (%)	6 (66,6)
	Mista n (%)	3 (33,3)
	Sensorioneural n (%)	0
Tipo de perda auditiva orelha contralateral	Condutiva n (%)	7 (77,7)
	Mista n (%)	2 (22,2)
	Sensorioneural n (%)	0
Média do limiar auditivo VO* (dBNA) orelha implantada	Média ± DP	9,2 ± 7,6
	Extensão	3,8 - 25
Média do limiar auditivo VO* (dBNA) orelha contralateral	Média ± DP	8,1 ± 5,5
	Extensão	2,5 - 20
Malformação OE e/ou OM	Etiologia não sindrômica n (%)	9 (100)
	Etiologia sindrômica n (%)	0

* Média do limiar audiométrico nas frequências de 500, 1k, 2k e 4 kHz

**Legenda**: VO = via óssea; dBNA = decibel nível de audição; OE = orelha externa; OM = orelha média; DP = Desvio padrão

A idade média no momento da ativação do processador de som foi de 24,3 anos (±10,5). Quanto ao tipo, seis indivíduos apresentaram perda condutiva na orelha implantada e a média dos limiares ósseos de 0,5 a 4 kHz variou de 3,8 a 7,5 dBNA; os demais apresentaram perda auditiva mista, cuja média variou de 8,8 a 25 dBNA.

É importante ressaltar que nenhum indivíduo deste estudo apresentou intercorrência pré ou pós-cirúrgica à implantação do componente interno e a ativação (programação) do processador de som, foi realizada após a avaliação e liberação da equipe médica. Em nosso serviço, a ativação é realizada pelo(a) fonoaudiólogo(a) no segundo mês após a implantação do componente interno, seguindo um protocolo de avaliação padronizado, o qual permite que as habilidades auditivas, bem como as necessidades individuais com relação à programação sejam verificadas longitudinalmente.

As configurações de programação do processador de som foram obtidas por meio do *software* Connexx (Sivantos), inserido na plataforma NOAH, a partir da realização do vibrograma – pesquisa dos limiares tonais nas frequências de 0,5 a 6kHz através do processador de som, o qual estimula diretamente o transdutor FMT. Para o ajuste do processador são utilizados os valores obtidos no vibrograma, bem como a preferência do indivíduo. A faixa de frequência do processador Amadè Hi se estende de 0,25 a 8 kHz, com ganho máximo de 54 dB, conforme apresentação do fabricante.

A Figura 3 apresenta a média dos limiares tonais em campo livre (dBNA) obtidos nas frequências de 0,5 a 4 kHz sem e com a prótese ativa de orelha média nos três momentos de avaliação: ativação, pós 1 e pós 2.

**Figura 3:** Média dos limiares tonais em campo livre obtidos nas frequências de 0,5 a 4 kHz sem e com a prótese ativa de orelha média nos três momentos de avaliação: ativação, pós 1 e pós 2. Significância estatística (p<0,05): estatisticamente significante para a condição sem prótese (a) com as condições ativação (b), pós 1(c) e pós 2 (d)

Legenda: dBNA = decibel nível de audição

A análise descritiva dos resultados dos limiares auditivos em campo livre revelou que, para o total de 9 indivíduos usuários unilaterais da prótese VSB (MED-EL), a média das frequências de 0,5 a 4 KHz foi de 51,7 dBNA (± 9,9) na condição pré cirúrgica. Em contrapartida, na condição ativação observou-se que a média foi de 25,7 dBNA (±5,1), correspondendo à melhora de 26 dBNA. No pós 1, a média foi 24,9 dBNA (± 5,3) e, no pós 2, a média estabeleceu-se em 23,8 (±5,0). Em análise, notou-se estabilização de resultados da média dos limiares auditivos em campo livre entre as condições ativação, pós 1 e pós 2.

Para a análise inferencial, os resultados mostraram melhora estatisticamente significante (p<0,05) já na ativação para os limiares tonais obtidos em campo livre na maioria das frequências (0,5 a 3 kHz), em relação à condição sem a prótese ativa de orelha média VSB (MED-EL) (Figura 3 e Tabela 2)^(25)^.

**Tabela 2:** Análise estatística ao comparar os limiares tonais em campo livre e percepção auditiva da fala sem e com a prótese ativa de orelha média nos três momentos de avaliação: ativação, pós 1 e pós 2

	0,5K	1K	2K	3K	4K	LRSS	S/R
Sem Prótese x Ativação	0,031*(a)	0,006*(a)	0,006*(a)	0,006*(a)	0,102	0,001*(a)	0,002**(a)
Sem Prótese x Pós 1	0,002*(b)	0,006*(b)	0,006*(b)	0,010*(b)	0,008*(b)	0,001*(b)	0,001**(b)
Sem Prótese x Pós 2	0,002*(c)	0,006*(c)	0,006*(c)	0,010*(c)	0,001*(c)	0,001*(c)	0,005**(c)
Ativação x Pós 1	0,844	1,000	1,000	0,998	0,798	0,832	0,999
Ativação x Pós 2	0,844	1,000	1,000	0,998	0,519	0,930	0,967
Pós 1 x Pós 2	1,000	1,000	1,000	1,000	0,968	0,995	0,932

* Teste Tukey (p<0,05) e Teste Brown-Forsythe (p<0,05)

Significância estatística (p<0,05): (a) estatisticamente significante - condição sem prótese x ativação para 0,5, 1, 2 e 3 kHz, LRSS e S/R: (b) estatisticamente significante - condição sem prótese x pós 1 para 0,5, 1, 2, 3 e 4 kHz, LRSS e S/R: (c) estatisticamente significante - condição sem prótese x pós 2 para 0,5, 1, 2, 3 e 4 kHz, LRSS e S/R

**Legenda**: LRSS = limiar de reconhecimento de sentenças no silêncio; S/R = relação sinal-ruído

Na Tabela 2 encontra-se a análise estatística ao comparar os limiares tonais em campo livre (dBNA) e percepção auditiva da fala sem e com a prótese ativa de orelha média nos três momentos de avaliação: ativação, pós 1 e pós 2.

Ao analisar a frequência de 4kHz, observou-se melhora entre o limiar auditivo observado na situação sem a prótese (55 dBNA ±9.7) e o momento da ativação (30 dBNA ±15.4); porém, diferença significante foi observada entre as situações sem a prótese e o pós 1, a qual se manteve estável no pós 2 (Figura 3 e Tabela 2). Este achado sugere melhor audibilidade após o período de estimulação auditiva.

Ademais, na Figura 3, foi possível observar que os limiares tonais obtidos em campo livre no momento da ativação da prótese mantiveram-se estáveis no pós 1 e no pós 2 para as frequências de 0,5 a 3 kHz.

A análise dos resultados com os testes de hipóteses aplicados na avaliação da percepção auditiva da fala no silêncio demonstrou melhora significante quando comparadas as situações sem a prótese e no momento da sua ativação (p<0,05). De modo similar ao observado com os limiares tonais em campo livre, após a ativação os resultados tornaram-se semelhantes e não apresentaram diferença significante (Figura 4 e Tabela 2).

**Figura 4:** Mediana dos limiares de reconhecimento de sentenças no silêncio (dBNA) sem e com a prótese ativa de orelha média nos três momentos de avaliação: na ativação, pós 1 e pós 2

Legenda: dBNA = decibel nível de audição; LRSS = limiar de reconhecimento de sentenças no silêncio

As Figuras 4 e 5 apresentam, respectivamente, a mediana dos limiares de reconhecimento de sentenças no silêncio (dBNA) e da relação S/R. A análise do LRSS revelou melhora de 28,5 dBNA na ativação e a relação S/R, observada em 3,2 dB no pré-cirúrgico, alcançou mediana de -2,5 dB na ativação. Importante ressaltar que, na avaliação de fala no ruído, quanto menor o valor da S/R, melhor o resultado obtido com o material de fala. Ao longo do tempo, os resultados obtidos nos testes de fala permaneceram estáveis, num nível igualmente significativo em comparação à condição pré-cirúrgica.

**Figura 5:** Mediana da relação S/R sem e com a prótese ativa de orelha média nos três momentos de avaliação: na ativação, pós 1 e pós 2

Legenda: dBNA = decibel nível de audição; S/R: relação sinal-ruído

## DISCUSSÃO

Com o avanço tecnológico ocorrido nos últimos anos, indivíduos com malformação de orelha externa e/ou média que estão impossibilitados ou não se beneficiam da amplificação por condução aérea, passaram a ter maiores opções de reabilitação auditiva no que se refere às próteses auditivas semi-implantáveis. Nessa categoria, se incluem a prótese auditiva de condução óssea e a ativa de orelha média.

A prótese ativa de orelha média Vibrant Soundbridge (MED-EL), objeto deste estudo, foi introduzida no mercado em 1996 e tem se destacado, uma vez que a sua indicação passou a abranger, além das perdas auditivas sensorioneurais, as condutivas e mistas^(26)^. Com as opções de acoplamento do transdutor interno (FMT), inicialmente restritos à cadeia ossicular, se estendendo para a janela oval e redonda, a prótese tornou-se uma possibilidade para indivíduos com malformação de orelha externa e/ou média^(22,27)^; porém, o possível local de acoplamento deve ser avaliado cuidadosamente por meio de exame de imagem realizado previamente, de modo que o cirurgião possa estimar o grau da malformação e determinar as possibilidades de acoplamento do FMT^(5)^.

O sucesso da cirurgia dependerá de uma conexão estável entre o transdutor e a estrutura anatômica que permita a vibração mecânica e a transmissão favorável do sinal, em todas as frequências, ao longo da cóclea^(5)^. Frente à segurança, efetividade e estabilidade do dispositivo observada em estudos longitudinais^(9,22,28,29)^, a prótese VSB (MED-EL) foi aprovada para crianças a partir de cinco anos^(13,15,16)^, o que possibilitou a inclusão de quatro participantes entre 12 e 17 anos neste estudo, nos quais não foi observada qualquer intercorrência pré ou pós-cirúrgica.

Para a avaliação audiológica do resultado da reabilitação auditiva fornecida pela VSB (MED-EL), parâmetros relacionados a audibilidade e percepção auditiva da fala precisam ser considerados e estabelecidos por meio de um protocolo clínico que favoreça o acompanhamento dos indivíduos quanto ao uso, benefício da tecnologia e necessidades de ajustes na programação.

Os limiares tonais observados em campo livre com a prótese mostraram excelente audibilidade para as principais frequências avaliadas logo no momento da ativação, que se estendeu em todos os momentos pós-cirúrgicos (Figura 1). Tais achados evidenciam que o acesso aos sons é possível pelo uso da prótese e favorece o resgate das habilidades de detecção de fala, essenciais para o desempenho nas tarefas de reconhecimento e compreensão da fala, e corroboram com estudos anteriores^(14,19,21)^.

Estudos anteriores^(14,21,27)^ corroboram a melhora dos limiares tonais com o uso de prótese ativa de orelha média logo no momento da ativação do processador e enfatizam que tais resultados são esperados devido à vibração das estruturas da orelha média proporcionada pelo posicionamento cirúrgico adequado do FMT e direcionamento do estímulo sonoro para a orelha interna^(11,20,21,28)^, fator esse que determina a importância de uma equipe médica bem treinada e experiente em cirurgias otológicas^(14)^.

A melhora observada na avaliação da percepção auditiva da fala por meio do limiar de reconhecimento de sentenças no silêncio logo na ativação e a estabilidade das respostas nas avaliações pós-cirúrgicas também apontou para efetividade da prótese (Figura 4 e Tabela 2). Estudos que avaliaram o desempenho do usuário com a prótese ativa de orelha média ao longo do tempo revelaram que a primeira mudança substancial nos resultados observados nos limiares tonais e nos limiares de reconhecimento de sentenças ocorreu até o terceiro mês^(9,18)^. Após esse período não foram observadas mudanças significativas^(14,16,28-30)^, indicando, assim, a estabilidade dos resultados.

A percepção auditiva da fala também foi avaliada na situação de ruído competitivo. Os resultados apontaram para o mesmo padrão observado na situação de silêncio; ou seja, o desempenho do indivíduo foi melhor logo na ativação do processador de som (Figura 5), com diferença significante (p<0,05), que se manteve estável nos demais momentos de avaliação, ou seja, no pós 1 e pós 2 (Tabela 2).

Sabe-se que resultados satisfatórios na percepção auditiva da fala estão relacionados ao melhor desempenho em situações de comunicação, principalmente em situações de ruído, condição inerente às situações da vida diária; o que tende a favorecer o uso efetivo do dispositivo auditivo e assim, proporcionar melhor qualidade de vida.

A estabilidade quanto aos limiares tonais obtidos em campo livre e na avaliação da percepção auditiva da fala realizados a curto e longo prazo foi verificada em estudos anteriores com a mesma prótese^(14,16,28-30)^. Essa estabilidade pode ser justificada pela aclimatização, pois as conexões atingem o pico de atividade máxima após estimulação em pouco tempo, com a otimização da prótese, obtendo assim o benefício desejado. A estimulação mecânica da orelha média tende a favorecer a plasticidade neural, permitindo que as vias auditivas centrais se reorganizem e proporcionem efeitos positivos nas habilidades auditivas^(11,20,21)^.

A avaliação do indivíduo na etapa pré-cirúrgica, na ativação da prótese e longitudinalmente, durante o acompanhamento, requer um protocolo previamente estabelecido pelo serviço de modo a avaliar, a audibilidade e a percepção auditiva da fala em situações de silêncio e ruído. O protocolo estabelecido em nosso serviço tem por objetivo estabelecer parâmetros para a comparação dos resultados ao longo do tempo, acompanhar a evolução do indivíduo e verificar as necessidades de ajustes do processador, de modo a garantir o uso efetivo e, assim, o alcance dos benefícios almejados.

Os resultados aqui apresentados estão conforme a literatura^(5,16,22,27-30)^, pois revelam melhora da audibilidade e da percepção auditiva da fala com a prótese, mesmo sem estimulação auditiva na orelha contralateral. A obtenção de resultados satisfatórios, observados longitudinalmente por meio de um protocolo clínico padronizado, sugere que a prótese ativa de orelha média VSB (MED-EL) é uma opção eficaz para indivíduos com malformação de orelha externa e/ou média, que não podem se beneficiar da reabilitação por meio do AASI por condução aérea. O uso efetivo da prótese tende a favorecer o processo de comunicação, socialização, desempenho acadêmico, inserção e manutenção no mercado de trabalho e, consequentemente, a melhora da qualidade de vida.

## CONCLUSÃO

Os resultados da prótese ativa de orelha média VSB (MED-EL) na audibilidade e na percepção auditiva da fala em indivíduos com malformação de orelha externa e/ou média foram melhores na condição da ativação, em comparação à condição pré-cirúrgica, mantendo-se estável ao longo do tempo; o que reforça sua indicação para essa população.
